# Diagnostic Impact of Subcutaneous Edema in Gouty Feet Detected by Dual-Energy Computed Tomography and Ultrasound

**DOI:** 10.3390/jcm13247620

**Published:** 2024-12-13

**Authors:** Julia Held, Christoph Strolz, Monique Reijnierse, Mihra Taljanovic, Pietro G. Lacaita, Miar Ouaret, Elke R. Gizewski, Günter Weiss, Andrea S. Klauser

**Affiliations:** 1 Department of Internal Medicine II, Medical University Innsbruck, Anichstrasse 35, 6020 Innsbruck, Austria; julia.held@tirol-kliniken.at (J.H.); guenter.weiss@i-med.ac.at (G.W.); 2 Department of Radiology, Medical University Innsbruck, Anichstrasse 35, 6020 Innsbruck, Austria; dr.strolz@meinradiologe.at (C.S.); miar.ouaret@i-med.ac.at (M.O.); elke.gizewski@i-med.ac.at (E.R.G.); andrea.klauser@i-med.ac.at (A.S.K.); 3 Division of Musculoskeletal Radiology, Department of Radiology, Leiden University Medical Center, 2333 ZC Leiden, The Netherlands; m.reijnierse@lumc.nl; 4 Departments of Medical Imaging and Orthopaedic Surgery, University of Arizona, Tucson, AZ 85724, USA; mihrat@radiology.arizona.edu; 5 Department of Radiology, University of New Mexico Health Sciences Center, Albuquerque, NM 87131, USA

**Keywords:** lymphedema, gout, gouty arthritis, DECT, ultrasound

## Abstract

**Background**: The objective of our study was to evaluate the association and frequency of subcutaneous lymphedema in patients with gout primarily affecting the feet. **Methods**: In 79 patients with acute gout, ultrasound (US) and dual-energy computed tomography (DECT) were performed to assess the presence of subcutaneous edema and extra- and intra-articular gouty deposits. In addition, the diagnostic utility of two post-processing DECT protocols were evaluated, comprising different minimum attenuation thresholds of 150 HU (DECT 150 protocol) and 120 HU (DECT 120 protocol), with the same maximum attenuation threshold (500 HU) and constant kilovoltage setting of tubes A and B at 80 and 140 kVp. **Results**: Subcutaneous lymphedema was present in 58.2% of patients, with a significant association with extra-articular monosodium urate (MSU) deposits (*p* < 0.001). Specifically, 97.8% of patients with lymphedema had extra-articular MSU deposits in DECT or US examination, while no cases of lymphedema were found in patients with exclusively intra-articular deposits. The DECT 120 protocol was significantly more sensitive for detecting peripheral MSU deposits (81%) compared to the DECT 150 protocol (34.2%, *p* < 0.001). **Conclusions**: Our findings demonstrate that the presence of lymphedema in patients with gout is frequently associated with extra-articular manifestations of the disease.

## 1. Introduction

Gout is an inflammatory disease characterized by the deposition of monosodium urate (MSU) crystals in joints, cartilage, and soft tissues and can lead to the formation of tophi [1]. Aspirate specimens and the demonstration of MSU crystals under polarizing microscopy are the gold standard for the diagnosis of gout [2,3]. However, this method is invasive and not always feasible, which has led to the increasing importance of ultrasound (US) and dual-energy computed tomography (DECT) in the diagnosis of gout [4,5,6,7,8,9].

Although gout is commonly associated with joint involvement, extra-articular manifestations are increasingly recognized, as supported by numerous studies [10,11,12,13,14,15,16].

These extra-articular presentations highlight the need for advanced imaging to better characterize gout beyond joint-specific involvement.

US is widely proposed as the first-line imaging modality due to its accessibility and ability to detect gout-specific features, such as the double contour sign and tophi [10]. However, extra-articular MSU deposits are often challenging to detect using US alone. DECT, by contrast, reliably identifies extra-articular MSU deposits, even in areas inaccessible to US, such as the cruciate ligaments of the knee. DECT also excels in differentiating MSU deposits from other pathologies, such as hydroxyapatite deposition disease [17]. Despite its diagnostic advantages, DECT is expensive and not universally available, underscoring the need to optimize more accessible modalities like US to assess extra-articular manifestations.

One intriguing but relatively underexplored aspect of extra-articular gout is its association with lymphedema, particularly of the feet. The prevalence of lymphedema in extra-articular gout of the feet varies across studies, ranging from sporadic case reports to small case series [18,19,20,21,22]. Clinical presentations may include unilateral or bilateral swelling, erythema, overheating, tenderness, and impaired lymphatic drainage. Lymphedema in gout is often localized to the dorsum of the foot, involving the metatarsophalangeal joints and extending proximally toward the ankle. The pathophysiological mechanisms underlying lymphedema in extra-articular gout remain incompletely understood. It is postulated that the chronic inflammation and tissue deposition of urate crystals lead to local tissue damage, the infiltration of inflammatory cells, the disruption of lymphatic channels, and impaired lymphatic drainage [23]. Additionally, comorbid conditions such as obesity, hypertension, and diabetes mellitus may contribute to lymphatic dysfunction and exacerbate tissue edema in patients with gout [24].

Existing studies from the literature have largely focused on the diagnostic capabilities of US and DECT for identifying intra-articular gouty deposits [16], while their role in assessing extra-articular manifestations, including subcutaneous lymphedema, remains underexplored. US is often considered the first-line imaging technique due to its accessibility and ability to detect gout-specific features such as the double contour sign and tophi. However, DECT provides a more comprehensive diagnostic overview, with the ability to reliably identify extra-articular MSU deposits, particularly in soft tissues.

This study aims to fill this gap by systematically evaluating the frequency and association of subcutaneous lymphedema with extra-articular MSU deposits in patients with acute gout attacks affecting the feet. By combining US and DECT imaging, we seek to identify the diagnostic value of these modalities in differentiating gout-associated lymphedema from other soft tissue conditions. Our findings could provide important insights for improving diagnostic accuracy and guiding clinical management, particularly in distinguishing gout-related edema from infections or other inflammatory conditions.

## 2. Materials and Methods

**Figure 1:**
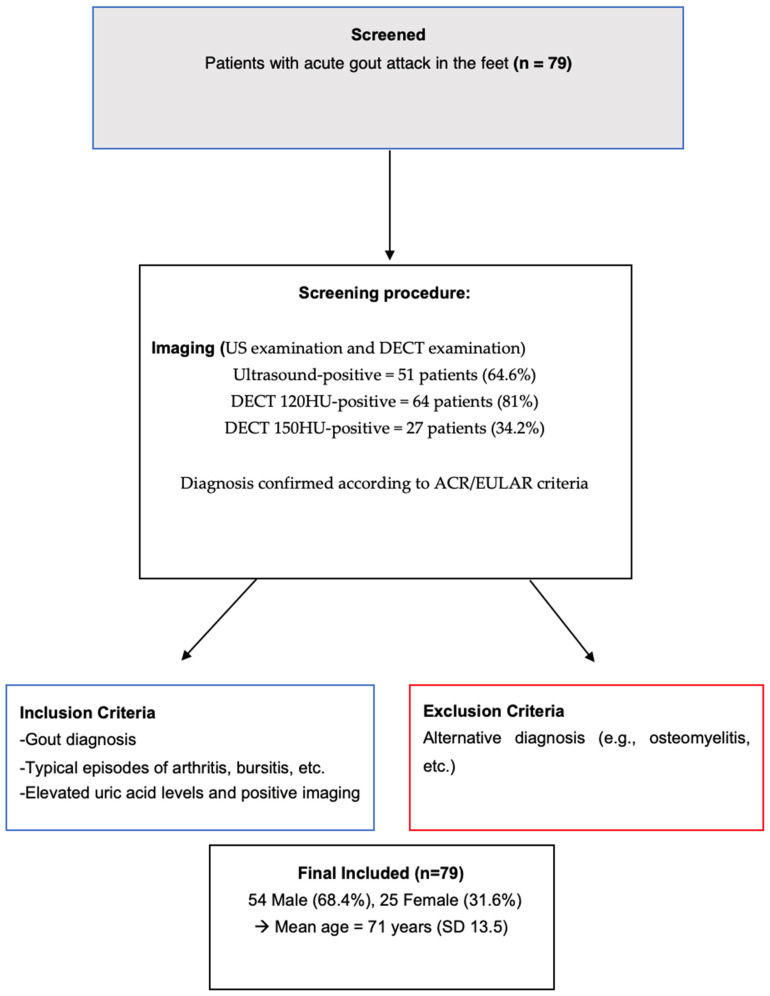
Patient flowchart.

A patient flowchart detailing the inclusion and exclusion criteria is shown in Figure 1.

### 2.1. Patients

All subjects gave their written informed consent for inclusion in the data analysis before they participated in the study. This study was conducted in accordance with the Declaration of Helsinki and approved by the Institutional Review Board of Medical University Innsbruck (AN2015-0209).

This study included 79 patients with acute gout of the feet who were referred to our department between October 2016 and March 2021 for radiological evaluation. The cohort was retrospectively analyzed, and no randomization was applied due to the observational nature of the study.

#### 2.1.1. Inclusion Criteria

Patients were included if they met the following criteria:

Diagnosed with gout according to the ACR/EULAR criteria, which requires at least one typical episode of peripheral arthritis, bursitis, pain, or tenderness.

Confirmed imaging findings of gout (US or DECT) or, in cases of negative imaging, the detection of urate crystals in synovial fluid or typical clinical presentation with a total score ≥ 8 according to the ACR/EULAR criteria [25].

#### 2.1.2. Exclusion Criteria

Patients were excluded if they presented with (1) alternative diagnoses such as osteomyelitis, cellulitis, or thrombosis or (2) incomplete clinical or imaging data.

### 2.2. Image Analysis

In our cohort, imaging analysis was performed to evaluate the presence of subcutaneous edema and MSU deposits using both ultrasound (US) and dual-energy computed tomography (DECT).

#### 2.2.1. Ultrasound Analysis

For subcutaneous edema, ultrasound findings were analyzed following the criteria established by Suehiro et al. [26], which define subcutaneous edema as areas with diffuse increases in echogenicity, identifiable horizontal or obliquely oriented echogenic lines from connective tissue bundles, and vertically oriented (≥45 degrees to the skin) echo-free spaces. Additionally, gout-specific ultrasound findings were assessed, including the following:

Double Contour Sign (DCS): An abnormal hyperechoic band along the superficial margin of the articular cartilage. This band could appear continuous or intermittent, irregular or regular, and can be differentiated from both the normal cartilage interface sign and calcium pyrophosphate deposits within the cartilage. Tophi are defined as circumscribed, inhomogeneous, hyperechoic, and/or hypoechoic aggregations that could generate posterior acoustic shadowing and are often surrounded by a small anechoic rim.

Aggregates and Tophi: Aggregates are defined as heterogeneous hyperechoic foci, while tophi are characterized as circumscribed, inhomogeneous, hyperechoic, or hypoechoic areas, sometimes with posterior acoustic shadowing.

#### 2.2.2. DECT Analysis

DECT was used to assess both intra- and extra-articular MSU deposits.

Two protocols were applied to enhance sensitivity: the DECT 120 protocol (with a minimum attenuation threshold of 120 HU) and the DECT 150 protocol (with a threshold of 150 HU). DECT is particularly valuable for identifying MSU deposits, even in the early stages of the disease, and provides incremental benefits when combined with other imaging techniques [27].

The findings included the following:

Extra-articular deposits: identified by green-coded regions on DECT color maps; extra-articular MSU deposits were found primarily in tendons and soft tissues.

Intra-articular deposits: intra-articular deposits were identified within joint spaces and cartilage.

### 2.3. Imaging Examination

#### 2.3.1. DECT Examination

DECT was performed using two Syngovia (Siemens, Erlangen, Germany) post-processing protocols with different minimum attenuation thresholds of 150 HU (DECT 150 protocol) and 120 HU (DECT 120 protocol), with the same maximum attenuation threshold (500 HU) and constant kilovoltage setting of tubes A and B at 80 and 140 kVp. The images were reconstructed using a software program (DE Gout; Siemens Helthineers) that provides color-coded images. A radiologist with six years of experience (CS) evaluated the color-coded DECT images and classified the results as positive or negative MSU deposits. Positive findings were defined as green-coded MSU deposits. Additionally, detailed assessments of MSU deposit locations differentiated between intra- and extra-articular deposits. Intra-articular MSU deposits were primarily identified within joint spaces and cartilage; they are also highlighted in green on the DECT images. In line with the American College of Rheumatology (ACR) and European League Against Rheumatism (EULAR) guidelines, nailbed, submillimeter, skin, motion, beam hardening, and vascular artifacts were not considered positive findings in our study [25].

#### 2.3.2. Ultrasound Examination

The sonographic examination followed the European Society of Musculoskeletal Radiology (ESSR) guidelines and was conducted using a 15–6 MHz linear array transducer (HI Vision Preirus, Hitachi Aloka Medical, Tokyo, Japan).

A positive ultrasound diagnosis was established based on the presence of a double contour sign (DCS), visible aggregates, or tophi and tendon deposits, following the OMERACT (Outcome Measures in Rheumatoid Arthritis Clinical Trials) guidelines. The DCS was identified as an abnormal hyperechoic band along the superficial margin of the articular hyaline cartilage, unaffected by the angle of insonation. This band could appear continuous or intermittent, irregular or regular, and was differentiated from both the normal cartilage interface sign and calcium pyrophosphate deposits within the cartilage. Tophi were defined as circumscribed, inhomogeneous, hyperechoic, and/or hypoechoic aggregations that could generate posterior acoustic shadowing and were often surrounded by a small anechoic rim. Aggregates, whether intra-articular or intratendinous, were identified as heterogeneous hyperechoic foci with high reflectivity, even under reduced gain settings or variable insonation angles, occasionally accompanied by posterior acoustic shadowing [28]. If none of these findings were observed, the ultrasound examination was considered negative.

### 2.4. Statistical Analysis

The absence/presence of lymphedema was further set in association with intra-/extra-articular gout manifestation as detected by US, DECT (120 and 150 HU) using an χ^2^-statistic based on a 2 × 2 tabulation. We applied the Mann–Whitney test for the comparison of age and lymphedema. Binary logistic regression was calculated to evaluate the effects of different parameters on lymphedema. All statistical analyses were performed using IBM SPSS (version 27). A *p*-value < 0.05 was used as the cut-off for statistical significance.

## 3. Results

### 3.1. Demographic and Clinical Characteristics

The study included 79 patients with acute gout in the feet, comprising 54 males (68.4%) and 25 females (31.6%) and a mean age of 71 (SD 13.5 years). Subcutaneous lymphedema was present in 58.2% (*n* = 46) of the patients. Age showed a significant association with the presence of lymphedema, as patients with lymphedema had a mean age of 75.5 years (SD 13.3) compared to 64.7 years (SD 11.3) in patients without lymphedema (*p* < 0.001). Table 1 summarizes the demographic characteristics and key imaging findings of the study cohort.

### 3.2. Imaging Results and Associations with Lymphedema

#### 3.2.1. Ultrasound Findings

**Figure 2:**
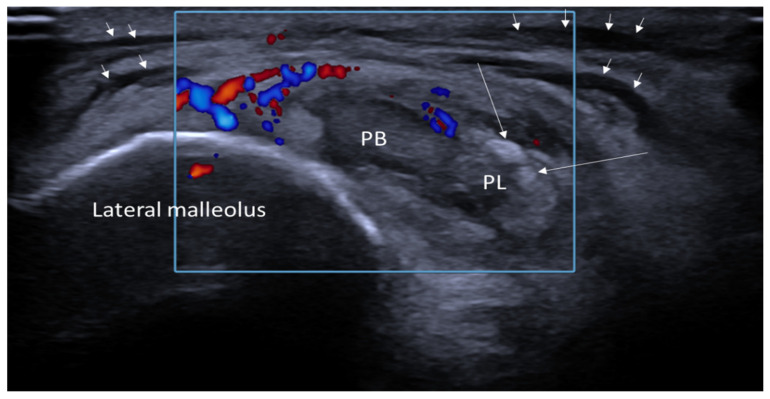
Axial ultrasound images of the ankle joint in a patient with gout, focusing on the peroneal tendons. The images reveal characteristic hyperechoic formations (long arrows), consistent with monosodium urate (MSU) deposits, representing an extra-articular manifestation of gout. Additionally, diffuse hypoechoic areas are visible in the subcutaneous tissue (short arrows), indicating the presence of lymphedema. PB: peroneus brevis tendon, PL: peroneus longus tendon.

Ultrasound findings were positive for gout-specific characteristics in 64.6% of patients (*n* = 51), including features such as the double contour sign, tophi, and aggregates. Positive ultrasound findings were strongly associated with the presence of lymphedema, with an odds ratio of 9.75 (*p* < 0.001). An example of a patient with an extra-articular manifestation of gout in US is illustrated in Figure 2, showing the characteristic US findings in lymph edema next to MSU deposits.

#### 3.2.2. DECT Findings

**Figure 3:**
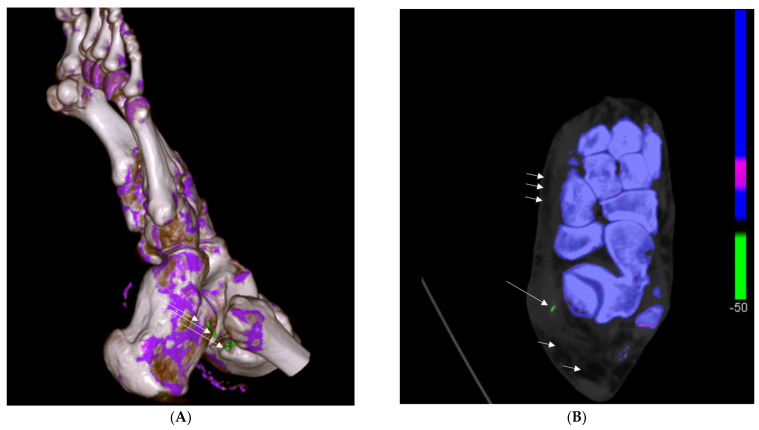
Dual-energy computed tomography (DECT) images of the same patient with extra-articular gout deposits at the peroneal tendon. (**A**): A 3D DECT reconstruction showing typical green-coded monosodium urate (MSU) deposits (long arrows) along the peroneal tendon. (**B**): Axial DECT scan of the ankle joint highlighting green-coded MSU deposits (long arrow) and areas of subcutaneous lymphedema (short arrows).

The DECT 120 HU protocol identified MSU deposits in 81% of patients (*n* = 64). The DECT 120 protocol showed higher sensitivity in detecting peripheral and extra-articular deposits compared to the DECT 150 HU protocol and resulted in a better association with the presence of lymphedema (*p* < 0.001 vs. *p* = 0.132, respectively; sensitivity for the DECT 120 protocol: 81%; sensitivity for the DECT 150 protocol: 34.2%). The DECT 150 HU protocol detected MSU deposits in 34.2% of patients (*n* = 27) but showed no significant association with lymphedema. An example of a patient with an extra-articular manifestation of gout in DECT is shown in Figure 3.

Among patients with lymphedema, 97.8% (*n* = 45 out of 46) presented extra-articular MSU deposits, reinforcing the link between lymphedema and extra-articular gout manifestations (*p* < 0.001). In contrast, neither of the two patients with exclusively intra-articular deposits exhibited lymphedema. Additionally, comorbid conditions such as obesity, hypertension, and diabetes mellitus were identified as potential contributors to lymphatic dysfunction, further exacerbating tissue edema in patients with gout (*p* < 0.001). Both DECT and ultrasound findings showed strong associations with the presence of lymphedema (*p* < 0.001 for both). Details of the main findings, including *p*-values, are presented in Table 2.

### 3.3. Binary Logistic Regression Analysis

Further analysis using binary logistic regression highlighted the significant predictive effect of imaging findings on the presence of lymphedema. Positive DECT 120 protocol findings showed the highest predictive strength, with an odds ratio of 16.6 (*p* < 0.001). Positive ultrasound findings also demonstrated a strong association with lymphedema (OR: 9.75; *p* < 0.001). The highest odds ratio was observed in patients with extra-articular MSU deposits, with an odds ratio of 43.3 (*p* < 0.001), emphasizing that extra-articular manifestations are a major factor in the development of lymphedema (details of the binary logistic regression analysis are illustrated in Table 3).

### 3.4. Clinical Significance of Statistical Findings

The odds ratios presented highlight the diagnostic utility of imaging techniques for identifying lymphedema in patients with gout. DECT 120 (OR: 16.6, *p* < 0.001) was particularly effective in detecting peripheral MSU deposits strongly linked to soft tissue involvement, while ultrasound (OR: 9.75, *p* < 0.001) remains a valuable first-line diagnostic tool. Furthermore, the superior sensitivity of the DECT 120 protocol proved critical in differentiating gout-associated lymphedema from other soft tissue inflammatory conditions, aiding in clinical decision-making. Extra-articular MSU deposits (OR: 43.3, *p* < 0.001) were the strongest predictor of lymphedema, underscoring their importance in the clinical evaluation of patients with gout.

## 4. Discussion

To our knowledge, this is the first study demonstrating the strong association of extra-articular MSU deposits and lymphedema in acute gout attacks. This finding could provide valuable insights for the treatment of future gout episodes, as the associated soft tissue reaction often leads to misdiagnosis; lymphedema, when combined with typical gout symptoms such as hyperemia and systemic inflammatory responses are frequently misinterpreted as local infections or cellulitis. Our data confirm the high sensitivity of DECT, particularly the 120 HU protocol, in detecting extra-articular MSU deposits linked to lymphedema. The DECT 120 protocol showed a sensitivity of 81% in identifying peripheral MSU deposits, significantly higher than the DECT 150 protocol, which had a sensitivity of 34.2%. This enhanced DECT 120 sensitivity was also significantly associated with the presence of lymphedema (*p* < 0.001), underscoring its clinical value for identifying gout-related tissue edema. By providing a reliable detection method for extra-articular manifestations, DECT 120 can aid in distinguishing gout from other soft tissue infections and inflammatory conditions that may present similarly.

Unfortunately, there are only a few studies describing extra-articular gout manifestations; thus, limited data on its prevalence are available. As no specific recommendation on optimal DECT protocols exists, we relayed an experimental protocol with a minimum attenuation threshold of 120 HU, which proved to be very sensitive in previous studies [16,29,30]. In our gout cohort, this high sensitivity was confirmed. Furthermore, binary logistic regression analysis supports the predictive role of DECT 120 in detecting lymphedema, with an odds ratio of 16.6 (*p* < 0.001) for positive findings, while the presence of extra-articular MSU deposits was associated with the highest odds ratio for lymphedema (OR = 43.3, *p* < 0.001). These results emphasize that extra-articular MSU deposits are key indicators of lymphedema risk in patients with gout and highlight the superior diagnostic potential of DECT 120 in managing these cases.

Our results suggest that the combined use of DECT and ultrasound enhances diagnostic accuracy, particularly for soft tissue involvement, providing clinicians with a more comprehensive view of gout-related changes. This combined approach could improve the differentiation between gout-associated lymphedema and other similar clinical presentations. Palazzi C et al. described a clinical case with the appearance of pitting edema in gout in the hand, distal forearms, and feet [31]. There was no further classification of gout into extra-articular or intra-articular forms. Additionally, Lu et al. [32] reported subcutaneous edema in up to 87.5% of patients with gout, with greater involvement of the lower extremities compared to the upper extremities. Yet again, no clear differentiation between intra- and extra-articular gout manifestation was made.

The OMERACT group provided clear definitions for intra- and extra-articular sonographic manifestations of gout. Their study found that up to 65% of patients with gout exhibited extra-articular manifestations in the patellar tendon, appearing as aggregates [33]. However, extra-articular gout without distinct aggregate accumulation may be missed in ultrasound examinations. To address these diagnostic challenges and gaps, particularly with extra-articular gout, DECT imaging, when used alongside conventional ultrasound, could aid in distinguishing gout from local infections.

Furthermore, this study opens avenues for additional research on the mechanisms linking gout and lymphedema. The pathways may involve lymphatic obstruction, inflammatory cytokines, or tissue damage induced by urate crystals, leading to localized edema and impaired lymphatic drainage. This would underscore the role of DECT as a highly sensitive tool for detecting gout-related tissue reactions and lymphedema, which may be further explored in prospective studies with larger cohorts.

As a limitation, this study did not include a control group for comparison, which reduces the generalizability of the findings. Future studies should incorporate control cohorts to validate the diagnostic utility of imaging techniques in differentiating gout-related lymphedema from other conditions. Additionally, another limitation of our study is the retrospective evaluation of the presence of edema. This could lead to false negative imaging results if the edema was not depicted in the stored pictures collected by the radiologist. Furthermore, the reported high sensitivity of the DECT 120 protocol needs to be evaluated in a cohort including control patients to define specificity. Lymphedema in the context of extra-articular gout of the feet represents a fascinating yet poorly understood clinical entity. While the exact prevalence and pathophysiology remain elusive, our findings suggest a strong link between gout and lymphedema. To implement these findings in future patient care, further prospective studies are needed to examine patients with gout more accurately and to better distinguish soft tissue infections from gout-associated lymphedema. Additionally, elucidating the underlying mechanisms of gout lymphedema warrants further research.

## 5. Conclusions

The presence of subcutaneous edema is a good indicator of the presence of extra-articular manifestations of gout. The significant predictive value of imaging modalities, especially DECT 120, highlights its critical role in identifying extra-articular deposits and differentiating gout-associated lymphedema from other conditions, such as infections or inflammatory diseases. These results underline the importance of advanced imaging techniques in improving diagnostic accuracy and guiding effective clinical management of gout-related complications.

## Figures and Tables

**Table 1:** Demographic characteristics and key imaging findings of the study cohort. This table summarizes the demographic characteristics and key imaging findings of the study cohort, including the prevalence of subcutaneous lymphedema (58.2%), positive ultrasound findings (64.6%), and the superior sensitivity of the DECT 120 HU protocol (81%) compared to DECT 150 HU (34.2%) in detecting MSU deposits. Extra-articular MSU deposits were more common (78.5%) compared to intra-articular deposits (30.4%). Ultrasound-positive usual findings of gout include tophi, aggregates, typical erosions, and double contour sign; 120 HU = DECT 120 protocol, 150 HU = DECT 150 protocol, MSU = monosodium urate deposits.

Demographics	
Gout diagnosis, *n* (%)	79 (100)
Age years, mean (SD)	71 (13.5)
Gender (female), *n* (%)	25 (31.6)
Lymphedema, *n* (%)	46 (58.2)
Ultrasound positive, *n* (%)	51 (64.6)
120 HU DECT positive, *n* (%)	64 (81)
150 HU DECT positive, *n* (%)	27 (34.2)
Extra-articular MSU deposits, *n* (%)	62 (78.5)
Intra-articular MSU deposits, *n* (%)	24 (30.4)
Only extra-articular MSU deposits, *n* (%)	40 (50.6)
Only intra-articular MSU deposits, *n* (%)	2 (2.5)

**Table 2:** Main findings of the study. This table presents the key findings of the study, including the prevalence of positive ultrasound results (64.6%) and extra-articular MSU deposits (78.5%). Older age was significantly associated with lymphedema (*p* < 0.001).

Findings	Lymphedema	No Lymphedema	*p*-Value
Ultrasound positive, *n* (%)	51 (64.6)	28 (35.4)	<0.001
Extra-articular MSU Deposits, *n* (%)	62 (78.5)	17 (21.5)	<0.001
Intra-articular MSU Deposits, *n* (%)	24 (30.4)	55 (69.6)	0.96
Age, years mean (SD)	75.5 (13.3)	64.7 (11.3)	<0.001

**Table 3:** Binary logistic regression predicting the presence of lymphedema. Positive ultrasound results showed an odds ratio of 9.75 (*p* < 0.001), indicating a strong association. Similarly, positive findings with the DECT 120 protocol yielded an odds ratio of 16.6 (*p* < 0.001), underscoring its predictive value for lymphedema.

Binary Logistic Regression		
	Odds Ratio	*p*-Value
Age	1.07	0.001
Gender	0.67	0.409
Ultrasound positive	9.75	<0.001
120 HU DECT positive	33.16	<0.001
150 HU DECT positive	2.19	0.118
Extra-articular MSU deposits	42.35	<0.001
Intra-articular MSU deposits	1.0	0.990

## Data Availability

The data presented in this study are available upon request from the corresponding author due to privacy reasons.

## References

[1] Girish G., Melville D.M., Kaeley G.S., Brandon C.J., Goyal J.R., Jacobson J.A., Jamadar D.A. (2013). Imaging appearances in gout. Arthritis.

[2] McQueen F.M., Chhana A., Dalbeth N. (2012). Mechanisms of joint damage in gout: Evidence from cellular and imaging studies. Nat. Rev. Rheumatol..

[3] Buckley T.J. (1996). Radiologic features of gout. Am. Fam. Physician.

[4] Dalbeth N. (2013). Management of gout in primary care: Challenges and potential solutions. Rheumatology.

[5] Glazebrook K.N., Guimarães L.S., Murthy N.S., Black D.F., Bongartz T., Manek N.J., Leng S., Fletcher J.G., McCollough C.H. (2011). Identification of intraarticular and periarticular uric acid crystals with dual-energy CT: Initial evaluation. Radiology.

[6] Bongartz T., Glazebrook K.N., Kavros S.J., Murthy N.S., Merry S.P., Franz W.B., Michet C.J., Veetil B.M.A., Davis J.M., Mason T.G. (2015). Dual-energy CT for the diagnosis of gout: An accuracy and diagnostic yield study. Ann. Rheum. Dis..

[7] Zhang Z., Zhang X., Sun Y., Chen H., Kong X., Zhou J., Zhou Y., Ma L., Jiang L. (2017). New urate depositions on dual-energy computed tomography in gouty arthritis during urate-lowering therapy. Rheumatol. Int..

[8] Finkenstaedt T., Manoliou A., Toniolo M., Higashigaito K., Andreisek G., Guggenberger R., Michel B., Alkadhi H. (2016). Gouty arthritis: The diagnostic and therapeutic impact of dual-energy CT. Eur. Radiol..

[9] Klauser A.S., Halpern E.J., Strobl S., Ellah M.M.H.A., Gruber J., Bellmann-Weiler R., Auer T., Feuchtner G., Jaschke W. (2018). Gout of hand and wrist: The value of US as compared with DECT. Eur. Radiol..

[10] Peiteado D., De Miguel E., Villalba A., Ordóñez M.C., Castillo C., Martín-Mola E. (2012). Value of a short four-joint ultrasound test for gout diagnosis: A pilot study. Clin. Exp. Rheumatol..

[11] Gerster J.C., Landry M., Rappoport G., Rivier G., Duvoisin B., Schnyder P. (1996). Enthesopathy and tendinopathy in gout: Computed tomographic assessment. Ann. Rheum. Dis..

[12] Weniger F.G., Davison S.P., Risin M., Salyapongse A., Manders E.K. (2003). Gouty flexor tenosynovitis of the digits: Report of three cases 1. J. Hand Surg..

[13] Primm D.D., Allen J.R. (1983). Gouty involvement of a flexor tendon in the hand. J. Hand Surg..

[14] Fernandes E.D., Sandim G.B., Mitraud S.A.V., Kubota E.S., Ferrari A.J.L., Fernandes A.R.C. (2010). Sonographic description and classification of tendinous involvement in relation to tophi in chronic tophaceous gout. Insights Imaging.

[15] Dalbeth N., Kalluru R., Aati O., Horne A., Doyle A.J., McQueen F.M. (2013). Tendon involvement in the feet of patients with gout: A dual-energy CT study. Ann. Rheum. Dis..

[16] Klauser A.S., Strobl S., Schwabl C., Kremser C., Klotz W., Nikodinovska V.V., Stofferin H., Scharll Y., Halpern E. (2023). Impact of Dual-Energy Computed Tomography (DECT) Postprocessing Protocols on Detection of Monosodium Urate (MSU) Deposits in Foot Tendons of Cadavers. Diagnostics.

[17] Schwabl C., Taljanovic M., Widmann G., Teh J., Klauser A.S. (2021). Ultrasonography and dual-energy computed tomography: Impact for the detection of gouty deposits. Ultrasonography.

[18] Shupper P., Stitik T.P. (2018). Tibialis Posterior Tenosynovitis: A Unique Musculoskeletal Manifestation of Gout. Am. J. Phys. Med. Rehabil..

[19] Scirocco C., Rutigliano I.M., Finucci A., Iagnocco A. (2015). Musculoskeletal ultrasonography in gout. Med. Ultrason..

[20] Bullocks J.M., Downey C.R., Gibler D.P.G., Netscher D.T. (2009). Crystal Deposition disease masquerading as proliferative tenosynovitis and its associated sequelae. Ann. Plast. Surg..

[21] Matcuk G.R., Katal S., Gholamrezanezhad A., Spinnato P., Waldman L.E., Fields B.K.K., Patel D.B., Skalski M.R. (2024). Imaging of lower extremity infections: Predisposing conditions, atypical infections, mimics, and differentiating features. Skelet. Radiol..

[22] De Vulder N., Chen M., Huysse W., Herregods N., Verstraete K., Jans L. (2020). Case Series: Dual-Energy CT in Extra-Articular Manifestations of Gout: Main Teaching Point: Dual-energy CT is a valuable asset in the detection of extra-articular manifestations of gout. J. Belg. Soc. Radiol..

[23] Ly C.L., Kataru R.P., Mehrara B.J. (2017). Inflammatory Manifestations of Lymphedema. International journal of molecular sciences. Int. J. Mol. Sci..

[24] Moffatt C.J., Gaskin R., Sykorova M., Dring E., Aubeeluck A., Franks P.J., Windrum P., Mercier G., Pinnington L., Quéré I. (2019). Prevalence and Risk Factors for Chronic Edema in U.K. Community Nursing Services. Lymphat. Res. Biol..

[25] Neogi T., Jansen T.L.T.A., Dalbeth N., Fransen J., Schumacher H.R., Berendsen D., Brown M., Choi H., Edwards N.L., Janssens H.J.E.M. (2015). 2015 Gout classification criteria: An American College of Rheumatology/European League Against Rheumatism collaborative initiative. Ann. Rheum. Dis..

[26] Suehiro K., Morikage N., Murakami M., Yamashita O., Ueda K., Samura M., Nakamura K., Hamano K. (2014). Subcutaneous tissue ultrasonography in legs with dependent edema and secondary lymphedema. Ann. Vasc. Dis..

[27] Lee S.K., Jung J.-Y., Jee W.-H., Lee J.J., Park S.-H. (2019). Combining non-contrast and dual-energy CT improves diagnosis of early gout. Eur. Radiol..

[28] Gutierrez M., Schmidt W.A., Thiele R.G., Keen H.I., Kaeley G.S., Naredo E., Iagnocco A., Bruyn G.A., Balint P.V., Filippucci E. (2015). International Consensus for ultrasound lesions in gout: Results of Delphi process and web-reliability exercise. Rheumatology.

[29] Strobl S., Kremser C., Taljanovic M., Gruber J., Stofferin H., Bellmann-Weiler R., Klauser A.S. (2019). Impact of Dual-Energy CT Postprocessing Protocol for the Detection of Gouty Arthritis and Quantification of Tophi in Patients Presenting with Podagra: Comparison with Ultrasound. Am. J. Roentgenol..

[30] Klauser A.S., Strobl S., Schwabl C., Klotz W., Feuchtner G., Moriggl B., Held J., Taljanovic M., Weaver J.S., Reijnierse M. (2022). Prevalence of Monosodium Urate (MSU) Deposits in Cadavers Detected by Dual-Energy Computed Tomography (DECT). Diagnostics.

[31] Palazzi C., Olivieri I., D’amico E., Pace-Palitti V., Petricca A. (2003). Symmetrical pitting edema resembling RS3PE in gout. Clin. Rheumatol..

[32] Lu C., Hsiao Y., Hsu H., Ko Y., Lin T., Chen L., Hsieh S., Li K. (2019). Can ultrasound differentiate acute erosive arthritis associated with osteomyelitis, rheumatoid arthritis, or gouty arthritis?. Int. J. Rheum. Dis..

[33] Terslev L., Gutierrez M., Christensen R., Balint P.V., Bruyn G.A., Sedie A.D., Filippucci E., Garrido J., Hammer H.B., Iagnocco A. (2015). Assessing Elementary Lesions in Gout by Ultrasound: Results of an OMERACT Patient-based Agreement and Reliability Exercise. J. Rheumatol..

